# Decoding Self vs. Non-Self: Alphavirus Cap0 Recognition and Immune Evasion

**DOI:** 10.3390/v18040439

**Published:** 2026-04-05

**Authors:** Santiago E. Faraj, Claudia V. Filomatori

**Affiliations:** 1 Departamento de Química Biológica, Facultad de Farmacia y Bioquímica, Universidad de Buenos Aires, Buenos Aires C1113AAD, Argentina; sefaraj@ffyb.uba.ar; 2 Instituto de Química y Fisicoquímica Biológicas “Prof. Alejandro C. Paladini” (IQUIFIB), Consejo Nacional de Investigaciones Científicas y Técnicas (CONICET)—Universidad de Buenos Aires, Buenos Aires C1113AAD, Argentina; 3 Cátedra de Biofísica y Bioestadística, Facultad de Odontología, Universidad de Buenos Aires, Buenos Aires C1122AAH, Argentina

**Keywords:** alphavirus, RNA, innate immune response, virus–host interactions, RIG-I, IFIT1

## Abstract

Host receptors can detect traces of non-self-pathogenic RNAs within a sea of cellular mRNA molecules. In host cells, mRNA cap methylation occurs in the nucleus, generating Cap1 and Cap2 structures (m^7^GpppNm and m^7^GpppNmNm, respectively). By contrast, alphavirus genomes carry a Cap0 structure (m^7^GpppN), which lacks 2′-O-methylation. This difference in the structure of the host and viral caps serves as a molecular signature that enables discrimination between self and non-self RNAs. Several host immune sensors, such as RIG-I and IFIT1, recognize the alphavirus Cap0 structure and trigger an antiviral response to restrict viral replication. It has been proposed that IFIT1 sequesters aberrant RNAs, preventing their translation by host ribosomes and blocking viral protein synthesis. However, alphaviruses have evolved molecular strategies to circumvent IFIT1-mediated restriction and facilitate infection in mammalian cells. One such strategy involves the folding of a 5′ RNA structure that hides the cap from host immune sensors. This highlights the dynamic interplay between viral evasion tactics and host immune defenses. This review will discuss how specific modifications at the 5′ end of alphavirus RNA modulate host defenses and how a deeper understanding of the virus–host interaction may inform the development of novel vaccine strategies.

## 1. Introduction

Alphaviruses are a global health threat due to their capacity to cause large-scale epidemics and severe disease in humans and animals [1,2]. Transmitted primarily by mosquitoes, these positive-sense, single-stranded RNA (ssRNA) viruses include several medically important pathogens. The Alphavirus genus is taxonomically organized into distinct antigenic complexes based on phylogenetic relationships, geographic distribution, and clinical manifestations [1,3]. Old World alphaviruses such as Semliki Forest virus (SFV), chikungunya virus (CHIKV), Mayaro virus (MAYV), and Ross River virus (RRV), are typically associated with arthritogenic disease. In contrast, New World alphaviruses are primarily encephalitogenic and are grouped into the Eastern, Western, and Venezuelan equine encephalitis complexes, which include eastern equine encephalitis virus (EEEV), western equine encephalitis virus (WEEV), and Venezuelan equine encephalitis virus (VEEV), respectively. Additionally, the genus encompasses a group of aquatic alphaviruses and a group of mosquito-specific alphaviruses, which do not infect vertebrates and display divergent biological properties.

Alphaviruses genomes are ~11–12 kb in length, capped at the 5′ end and polyadenylated at the 3′ end. They contain two open reading frames (ORFs): the first encodes four non-structural proteins (nsP1–nsP4) involved in RNA replication [4], while the second encodes six structural proteins (capsid, E1, E2, E3 and 6K/TF) that form the viral particle [1]. These ORFs are flanked by the 5′ and 3′ untranslated regions (UTRs), as well as an internal UTR that includes the subgenomic promoter. Although untranslated, these regions harbor cis-acting elements that play important regulatory roles [5].

In recent decades, alphaviruses have reemerged, with increasing frequency and geographic spread of outbreaks [6]. Historically confined to specific ecological niches, several alphaviruses have expanded their range due to a combination of ecological, climatic, and anthropogenic factors. For example, CHIKV, once limited to Africa and Asia, caused major epidemics in the Indian Ocean region in the mid-2000s and later spread to the Americas in 2013, resulting in millions of infections [7].

Despite their global impact, vaccine options are limited and available for only a few of these viruses, and no specific antiviral therapies have been approved. This underscores the urgent need to better understand the mechanisms governing virus–host interactions, including host antiviral responses and the viral strategies that subvert them. In this review, we summarize current knowledge on how host cells recognize alphavirus RNA and how specific viral RNA structures enable evasion of host RNA-sensing mechanisms.

## 2. Antiviral Innate Immune Response

**Figure 1:**
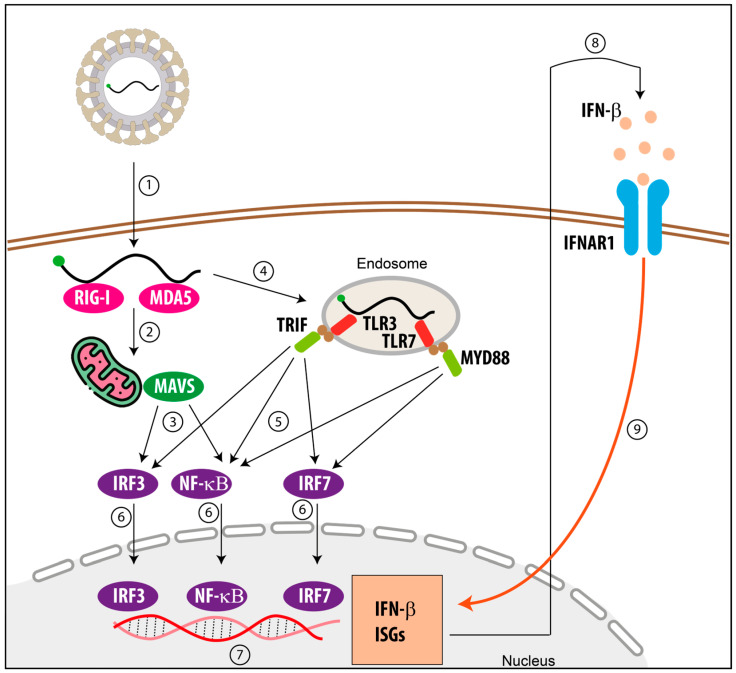
Simplified schematic representation of innate immune signaling triggered by RNA virus infection. In the cytoplasm, RIG-I-like receptors (RLRs), such as melanoma differentiation-associated protein 5 (MDA5) and retinoic acid-inducible gene I (RIG-I), recognize viral RNA species ① and signal through the mitochondrial antiviral signaling protein (MAVS) ②, leading to activation of IFN regulatory factor 3 (IRF3) and nuclear factor κB (NF-κB) ③. In parallel, endosomal Toll-like receptors (TLRs), including TLR3 and TLR7, detect viral nucleic acids ④, and signal through the adaptor proteins TRIF or MYD88, respectively ⑤. These pathways converge on the activation of IRF3, IRF7, and NF-κB, which translocate to the nucleus ⑥ to promote transcription of interferon-β (IFN-β) and other pro-inflammatory genes ⑦. Secreted IFN-β then binds to the type I IFN receptor (IFNAR1) in an autocrine and paracrine manner ⑧, triggering downstream signaling cascades that lead to the expression of IFN-stimulated genes (ISGs) ⑨. Numbered steps indicate sequential signaling events and amplification loops (red arrow) within the innate immune response.

As soon as an RNA virus enters the host cell, its genomic material and replication intermediates are rapidly sensed by the innate immune system. These viral features, collectively termed pathogen-associated molecular patterns (PAMPs), include double-stranded RNA (dsRNA), uncapped 5′-triphosphate termini, and highly structured ssRNAs [8]. Host cells detect PAMPs through specialized pattern recognition receptors (PRRs), including melanoma differentiation-associated gene 5 (MDA5) and retinoic acid inducible gene I (RIG-I) in the cytoplasm, as well as Toll-like receptors (TLRs) localized within endosomal compartments (Figure 1) [2,9,10]. Recognition of viral RNA by PRRs initiates signaling cascades that activate transcription factors such as IRF3, IRF7, and NF-κB. This ultimately results in the production of pro-inflammatory cytokines and type I interferons (IFNs) like IFN-β.

Type I IFNs signal through a common interferon receptor (IFNAR1) in both autocrine and paracrine fashions [11], triggering downstream pathways that induce the expression of hundreds of interferon-stimulated genes (ISGs). Between 500 and 1000 genes can be upregulated, depending on the cell type and physiological context [12]. The products of these ISGs establish an antiviral state that restricts viral replication and shapes subsequent adaptive immune responses. Notably, the IFN response is self-reinforcing, amplifying both the magnitude and spatial spread of antiviral signaling.

## 3. mRNA Cap Modifications Define Self vs. Non-Self RNA

**Figure 2:**
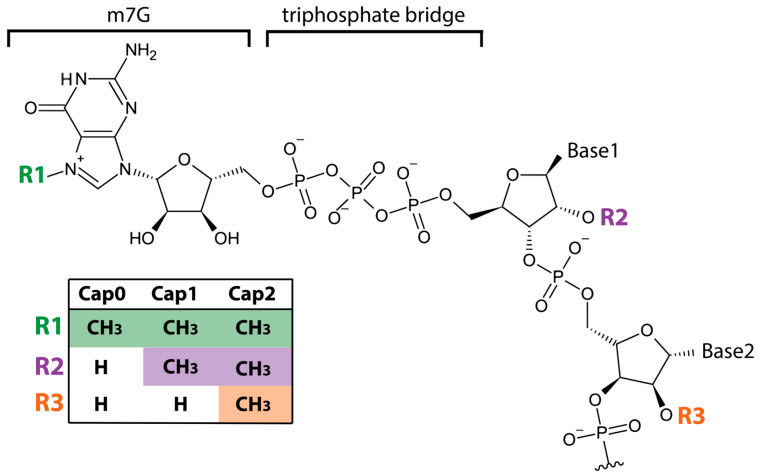
Schematic representation of the Cap0, Cap1, and Cap2 RNA structures. The Cap consists of a 7-methylguanosine (m^7^G) moiety linked to the first transcribed nucleotide via an unusual 5′-5′ triphosphate bridge (5′ppp). In the Cap0 structure, methylation is present only at the N7 position of the guanine cap. Cap1 is generated by an additional 2′-O-methylation of the ribose of the first nucleotide (R2), whereas Cap2 includes a further 2′-O-methylation at the ribose of the second nucleotide (R3). The 2′-O-methylations are typical of eukaryotic mRNAs and allow the host to distinguish self- from non-self mRNAs.

mRNA capping occurs co-transcriptionally in the nucleus through a series of enzymatic reactions that generates a 7-methylguanosine residue linked to the first nucleotide of the transcript via a 5′-5′ triphosphate bridge (m^7^GpppN, Cap0) (Figure 2). This structure serves essential functions: it protects the mRNA from exonucleolytic degradation, promotes nuclear export, enhances translation initiation through recognition by eukaryotic factor eIF4E, and contributes to RNA quality control mechanisms such as nonsense-mediated decay [13,14,15] (for review see Refs. [16,17]).

It is well established that beyond their roles in RNA stability and translation, cap modifications are critical determinants of self- versus non-self RNA discrimination. In higher eukaryotes, the cap structure undergoes additional 2′-O-methylation at the ribose of the first nucleotide, or both the first and second nucleotides, generating Cap1 and Cap2 structures, respectively (Figure 2) [18,19]. These modifications function as molecular signatures of self RNA [20], whereas many RNA viruses, including alphaviruses, retain a 2′-O-unmethylated cap structure (Cap0) at the 5′ end of their genomes.

Several components of the host innate immune system selectively recognize Cap0 RNAs and mount antiviral responses to infection.

## 4. Alphavirus RNA Sensing by the Innate Immune Receptor RIG-I

It is known that RIG-I-like receptors, including RIG-I and MDA5, possess a remarkable ability to distinguish cellular self-RNAs from pathogenic non-self RNAs (Figure 1) [21,22,23]. However, these receptors recognize distinct RNA signatures [24,25]. Although both RIG-I and MDA5 primarily detect dsRNA, only RIG-I is highly sensitive to the chemical and structural features of the RNA 5′ termini [26,27].

**Figure 3:**
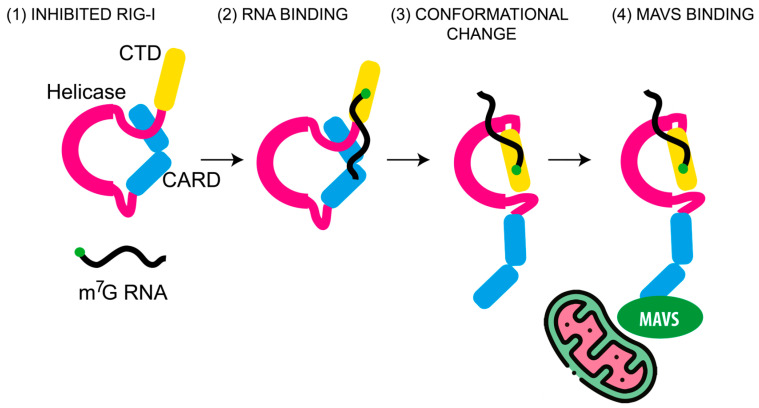
Model of RIG-I interaction with non-self RNA. Schematic representation of stepwise activation based on experimental findings [27,28]. In its basal state (1), RIG-I adopts an autoinhibited conformation in which the N-terminal CARD domains are masked through intramolecular interactions with the helicase (in magenta) and C-terminal domain (CTD, in yellow), preventing downstream signaling. The CTD binds to non-self RNA, typically short blunt-ended dsRNA bearing a 5′ppp or a 5′Cap0 structure (2), inducing a conformational change that exposes the N-terminal caspase activation and recruitment domains (CARDs, in light blue) (4). The exposed CARDS then become ubiquitinated and promote MAVS aggregation at the mitochondrial membrane.

RIG-I belongs to the DExH/D-box family of RNA helicases and contains three central helicase domains flanked by a regulatory C-terminal domain (CTD) and two N-terminal caspase activation and recruitment domains (CARDs) (Figure 3). The helicase domains, together with the CTD, mediate RNA binding, while the CARDs initiate downstream signaling through interaction with adaptor proteins.

In the absence of RNA ligands, RIG-I adopts an inactive, autoinhibited, closed conformation. Binding to an RNA PAMP allows the helicase domain to encircle the RNA, inducing a conformational rearrangement that exposes the CARDs [28]. These domains interact with the Mitochondrial Antiviral Signaling protein (MAVS) located on the mitochondrial membrane, thereby initiating antiviral signal cascades (Figure 1 and Figure 3). Cryo-electron microscopy studies have revealed that RIG-I adopts distinct conformational states depending on the nature of the RNA 5′ terminus [27].

Notably, Cap0 and 5′-triphosphate (5′ppp) RNAs bind RIG-I with nearly identical dissociation constants (Kd ≈ 1.7 and 1.8 nM, respectively) and induce comparable RIG-I’s ATPase activity and cellular signaling responses [26]. Efficient binding requires a short-blunt-ended dsRNA structure, as Cap0 and 5′ppp ssRNAs fail to bind RIG-I and do not trigger signaling. RIG-I recognition of viral dsRNA is length-dependent: short dsRNA species (<500 bp) promote efficient signaling activation, whereas long dsRNA (>500 bp) forms more stable complexes that are less effective at initiating downstream signaling [29]. Importantly, RIG-I binds to eukaryotic Cap1 RNAs with very low affinity (Kd > 400 nM), indicating that 2′-O-unmethylated RNAs serve as molecular signatures of non-self RNA [26]. A recent study further demonstrated that accumulation of Cap1 RNAs can also lead to RIG-I activation, particularly under conditions of elevated RIG-I expression. To prevent aberrant activation, long-lived host mRNAs undergo an additional methylation step that converts Cap1 to Cap2, thereby reducing their capacity to bind and activate RIG-I [30].

Collectively, these findings highlight the exquisite sensitivity of RIG-I to the structural features of the RNA 5′ termini and underscore its central role in distinguishing self from non-self RNA, enabling selective antiviral responses against alphavirus genomes.

## 5. Cap-Dependent Sensing and Translational Control by IFIT1

The interferon-induced proteins with tetratricopeptide repeats (IFITs) were among the first ISGs to be discovered nearly four decades ago [31]. IFIT homologs are evolutionarily conserved from amphibians to mammals [32]. In humans, four members of this family have been well characterized: IFIT1 (also known as ISG56), IFIT2 (ISG54), IFIT3 (ISG60) and IFIT5 (ISG58). Under basal conditions, IFIT proteins are only minimally expressed in most cell types, with the exception of specific subsets of myeloid cells [33]. However, their transcription is rapidly and robustly induced following viral infection [34] or stimulation with exogenous type I IFNs [35].

IFIT proteins are cytoplasmic and lack intrinsic enzymatic activity. Instead, they contain multiple tetratricopeptide repeat motifs (TPRs), the number of which varies among family members [34]. These TPR domains mediate protein–protein and protein–RNA interactions [36,37,38,39,40,41,42], enabling IFITs to inhibit the replication of diverse viruses through distinct mechanisms.

**Figure 4:**
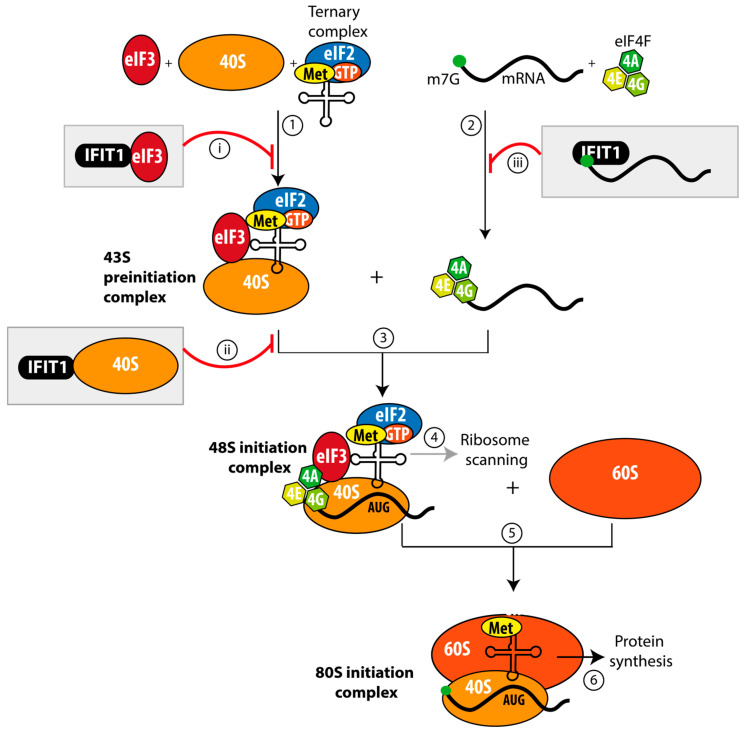
Mechanism of translation inhibition by IFIT1. Schematic representation of cap-dependent translation initiation in eukaryotic cells and its inhibition by the interferon-stimulated protein IFIT1. Under normal conditions, the 40S ribosomal (in light orange) subunit associates with eIF3 (in wine) and the ternary complex (eIF2-GTP-Met-tRNA) to form the 43S preinitiation complex ① [45,46]. In parallel, the eIF4F cap-binding complex (composed of eIF4A, eIF4E and eIF4G; in green) binds to the 5′ m^7^G cap structure ② and recruits the 43S preinitiation complex to form the 48S preinitiation complex ③. Once assembled, the 48S complex scans along the mRNA to locate the AUG start codon ④, where GTP hydrolysis and factor release promote joining of the 60S ribosomal subunit (in dark orange) to form the 80S initiation complex ⑤, allowing productive protein synthesis ⑥. IFIT1 (in black) inhibits RNA translation through multiple mechanisms (indicated by red lines): 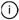 it binds to the eIF3 complex, preventing 43S preinitiation complex assembly [16,17,18], 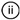 it interacts with the 40S ribosomal subunit, blocking 48S complex formation [43]; and 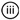 it directly binds to Cap0 viral RNA, preventing the recruitment of the eIF4F complex [43,44].

Among the IFIT family members, IFIT1 is the best characterized. It impairs cap-dependent translation through multiple mechanisms (Figure 4). Structural studies have well established that IFIT proteins possess positively charged grooves capable of accommodating the m^7^G cap and the triphosphate bridge via electrostatic interactions with conserved lysine and arginine residues, while the guanine moiety is further stabilized by stacking interactions within the binding pocket [39,40]. Similar to RIG-I, IFIT1 binds to RNA in a manner dependent on its 5′ capping status. Proteomic analyses combined with functional validation demonstrated that IFIT1 preferentially recognizes Cap0 over 2′-O-methylated caps [36,37,38,39]. At the molecular level, the 2′-hydroxyl groups (2′-OH) of the ribose contribute to RNA–protein interactions in a way that depends on the sugar pucker. In Cap0 RNAs, the unmethylated 2′-OH is compatible with the IFIT1 binding pocket. In contrast, 2′-O-methylation in Cap1 structures alters the local geometry and chemical properties of the ribose, reducing binding affinity [43,44]. The base at position 1 is stabilized by van der Waals contacts and stacking interactions but does not appear to form specific hydrogen bonds with the protein, allowing flexibility in base recognition. Consistent with this, downstream nucleotides interact with flexible regions of the protein, indicating that IFIT proteins can accommodate a broad range of 5′-terminal RNA sequences. Overall, RNA binding is primarily driven by shape complementarity and non-sequence-specific interactions. Notably, nucleotides immediately downstream of the Cap0 structure can influence binding efficiency; although RNA is bound in a non-sequence-specific manner, it requires a 5′ overhang of approximately three nucleotides [37].

Ultimately, IFIT1 sequesters alphavirus genomes, preventing their association with eukaryotic translation initiation factors and thereby restricting productive viral translation.

In addition to directly targeting viral RNA, IFIT1 has been proposed to interfere with host translation machinery, removing translation factors from the active pool. For example, IFIT1 targets human eukaryotic translation initiation factor 3 (eIF3), disrupting its association with the ternary complex and thereby preventing assembly of the 43S pre-initiation complex [16,17,18]. Furthermore, IFIT1 may associate directly with the 40S ribosomal subunit, interfering with the formation of the 48S initiation complex [43] (Figure 4).

In summary, IFIT1 restricts translation by targeting both viral RNA and components of the host translation machinery. While recognition of alphavirus Cap0 structures accounts for the selective inhibition of viral protein synthesis, interactions with host translation factors likely contribute to the broader translational shutoff observed during infection.

## 6. A Viral RNA Structure Avoids Host Recognition of Alphavirus RNA

Viruses have evolved multiple strategies to counteract host immune responses, and alphaviruses are no exception (for review see Ref. [47]). The first nucleotides of the alphavirus genomes are predicted to fold into a conserved 5′ stem-loop (5′SL) structure [48,49,50], which has been proposed to limit accessibility of the Cap0 structure to IFIT1 [49,51,52]. As noted above, cap accommodation within the IFIT1 binding pocket requires a ssRNA overhang. In this context, base-pairing at the 5′ end constrains the RNA into a conformation that may be incompatible with insertion into the IFIT1 binding channel, thereby impairing cap recognition. Consequently, the 5′SL has been suggested to act as a structural shield that functionally mimics Cap1-mediated evasion. Consistent with this model, despite having a Cap0 structure, alphavirus RNAs remain efficiently translated even in cells expressing IFIT1 [43].

This potential immune evasion strategy was first characterized in VEEV and its attenuated vaccine strain TC-83, which differs from the virulent Trinidad donkey (TRD) strain by two mutations [53]. One of these mutations, a G3A substitution in the 5′UTR, has been suggested to disrupt proper 5′SL formation [54]. In a key study, Hyde et al. [52] compared infection outcomes in wild-type (WT) and *Ifit1−/−* mice. While both mouse genotypes were equally susceptible to the virulent TRD strain, TC-83 was attenuated in WT animals but caused lethal disease and higher viral loads in *Ifit1−/−* mice. These findings support a role for IFIT1 in restricting the attenuated TC-83 strain, but not the virulent TRD strain.

Complementary experiments using chimeric VEEV/SINV viruses differing only at nucleotide position 3 of the genome (G3 versus A3) further support the importance of the 5′UTR in modulating IFIT1 sensitivity [52]. Upon infection of the IFN-treated mouse embryonic fibroblasts (MEFs), the mutant A3 variant was highly sensitive to IFIT1 restriction and failed to replicate in WT cells but was partially rescued in *Ifit1−/−* MEFs. These results suggest that even single-nucleotide changes within the 5′UTR can influence IFIT1 sensitivity and viral fitness.

Similar observations have been described for other alphaviruses. Mutations predicted to disrupt the 5′SL in SINV and CHIKV also result in viral attenuation [52,55]. However, the extent of IFIT1 sensitivity appears to vary across the genus. For example, EEEV strains from both North and South America show strong inhibition by IFIT1, whereas viruses such as SINV (AR339), CHIKV (181/25), and VEEV (3908) display relatively higher intrinsic resistance [52,55]. Notably, viruses that exhibit only partial resistance to IFIT1, such as EEEV, are likely compensated by the low levels of type I IFN induced in cultured cells and animal models [56]. Differences in IFIT1 sensitivity may reflect variations in the stability, dynamics, or topology of the 5′SL. However, this relationship is not yet fully established and may be influenced by additional factors.

Taken together, the model of cap shielding by the 5′SL has been proposed for selected alphaviruses; however, its conservation across the entire genus remains to be established.

## 7. Conservation Analysis of Alphavirus 5′UTRs and Structure of Their 5′SLs

**Figure 5:**
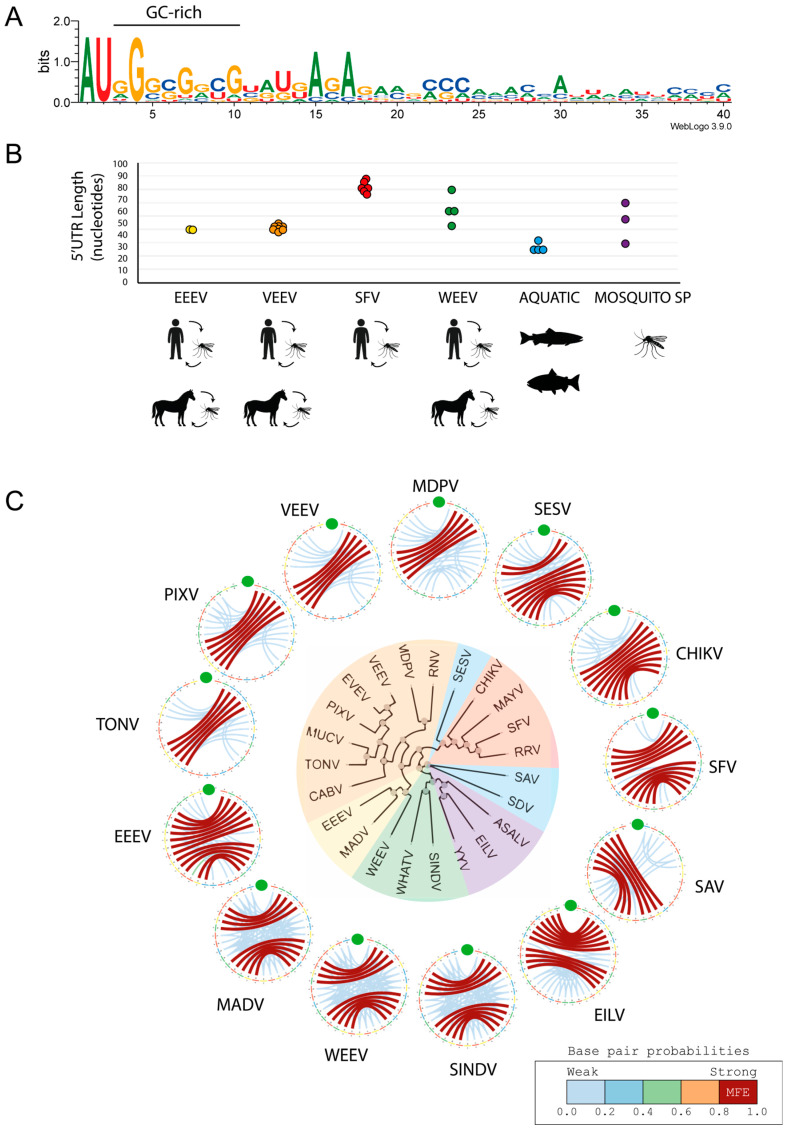
Comparative sequence and structural analysis of the 5′UTR across alphavirus ecological groups. (**A**) Sequence logo representation of nucleotide conservation within the first 40 nucleotides of the viral genome from representative alphaviruses. (**B**) Length distribution of the 5′UTRs across alphavirus ecological groups, each represented by a different color. (**C**) Fan dendrogram of representative alphaviruses. Color shading indicates alphavirus complexes as follows: Eastern equine encephalitis complex: eastern equine encephalitis virus (EEEV, KJ469568.1) and Madariaga virus (MADV, KJ469626.1); Venezuelan equine encephalitis complex: Venezuelan equine encephalitis virus (VEEV, AY823299.1), Everglades virus (EVEV, NC_038671.1), Pixuna virus (PIXV, NC_038673.1), Mucambo virus (MUCV, AF075253), Tonate virus (TONV, NC_038675.1), Cabassou virus (CABV, NC_038670.1), Rio Negro virus (RNV, NC_038674.1), and Mosso das Pedras virus (MDPV, NC_038857.1); Mosquito-specific virus complex: Agua Salud virus (ASALV, MK959114.1), Yada Yada virus (YYV, MN733821.1), and Eilat virus (EILV, NC_018615.1); Western equine encephalitis complex: Sindbis virus (SINV, NC_001547.1), Whataroa virus (WHATV, NC_016961.1), and western equine encephalomyelitis virus (WEEV, NC_003908.1); Semliki Forest complex: chikungunya virus (CHIKV, KF318729.1), Semliki Forest virus (SFV, NC_003215.1), Ross River virus (RRV, GQ433354.1), and Mayaro virus (MAYV, NC_003417.1); Aquatic virus complex: southern elephant seal virus (SESV, NC_016960.1), salmonid alphavirus (SAV, JQ799139.1), and sleeping disease virus (SDV, NC_003433.1). The scale bar indicates the number of nucleotide substitutions per site. Circular plot of base-pairing probabilities in representative alphaviruses. Base-pairing probabilities are color-coded from weak (in light blue) to strong (in wine), and arcs denote predicted base pairs. The Cap0 structure is highlighted with green circles. Structures were generated using Circos version 0.69–3 and RNAfold version 2.4.6 from the ViennaRNA Package version 2.0.

Sequence analysis of alphavirus genomes reveals limited overall conservation within the 5′UTR (Figure 5A). Notably, despite this variability, most genomes retain an AU dinucleotide proximal to the 5′ cap, followed by a GC-rich region immediately downstream, suggesting potential functional constraints at the 5′ end. Although the length of the 5′UTR is relatively conserved within each alphavirus complex, it varies considerably among members of different complexes (Figure 5B). The 5′UTR length ranges from ~30 nucleotides in aquatic alphaviruses to ~40 nucleotides in the VEEV and EEEV complexes, and up to ~80 nucleotides in the SFV complex. In contrast, WEEV and mosquito-specific complexes 5′UTRs are more heterogeneous, ranging from 48 to 77 and 33 to 67 nucleotides, respectively.

Despite limited primary sequence conservation, nucleotides immediately downstream of the Cap0 structure engage in base-pairing interactions, suggesting that this region may contribute to the folding of an RNA structural element (Figure 5C).

**Figure 6:**
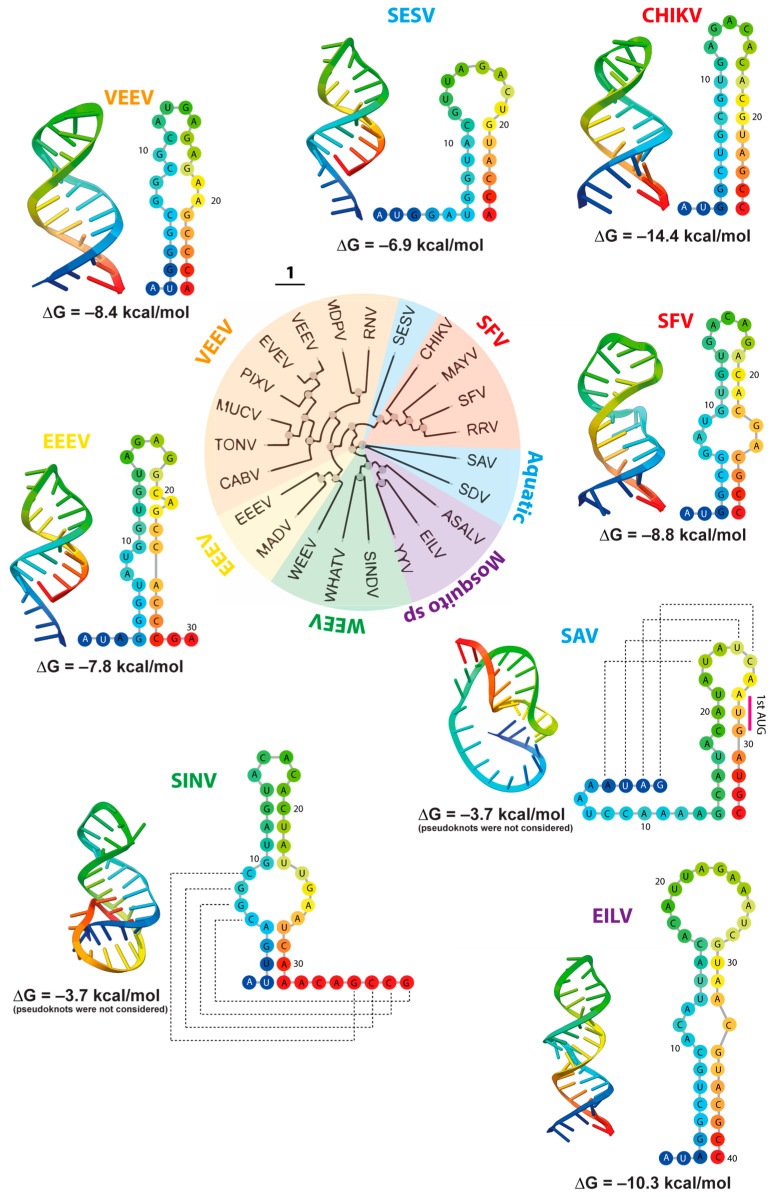
Representation of 5′ RNA structure similarities in alphaviruses. Fan dendrogram of representative alphaviruses from different viral complexes, each shown in a different color. The first nucleotides of each viral genome were folded using the Alphafold algorithm to predict 3D structures. Based on these predictions, RNA 2D structures were modeled with forna. Both 2D and 3D representations are shown using a rainbow color scale, which indicates nucleotide position along the RNA, with the 5′ end depicted in blue and the 3′ end in red. Thermodynamic stability of each predicted structure was estimated with the RNAeval program. For SINV and SAV, the calculated free energies (ΔG) are underestimated, as the program does not account for stabilization mediated by pseudoknot interactions (dashed lines).

To gain insight into the three-dimensional (3D) structure of this element, representative 5′SLs from distinct alphavirus complexes were modeled and compared (Figure 6). Recent advances in machine learning and artificial intelligence tools, originally developed for protein structure prediction, are increasingly being adapted for RNA structural modeling [57,58]. Using the AlphaFold algorithm, we generated predicted 3D models of the alphavirus 5′SLs and subsequently converted them into two-dimensional (2D) representations using the forna RNA visualization program [59] (Figure 6).

To assess the quality of the AlphaFold structural models, we analyzed Predicted Aligned Error (PAE) matrices, which estimate the positional error between pairs of nucleotides. PAE analyses showed low error values along the diagonal, supporting high confidence in local structural features, but increased off-diagonal values, indicating reduced certainty in long-range interactions (Figure S1A). This pattern is consistent with current limitations in AlphaFold’s ability to predict RNA structure: while it performs well in capturing local geometry, it is not specifically calibrated to resolve global RNA tertiary organization [60]. In agreement with this, mean per-residue predicted Local Distance Difference Test (pLDDT) values showed median scores around 80 across the analyzed 5′SL elements (Figure S1B). These values indicate high internal consistency of nucleotide geometry. Notably, RNAs with less well-defined structural organizations (e.g., SINV and SAV) exhibit lower pLDDT values and higher off-diagonal PAE values.

Importantly, the predicted stem-loop architectures are consistent with SHAPE reactivity and functional data [48,49,61], as well as RNAfold predictions (Figure 5), supporting the canonical structural organization of the alphavirus 5′SL. Accordingly, these models are used as structural representations compatible with experimentally supported secondary structure, rather than as de novo predictors of global RNA folding.

First, we analyzed the 5′SL of mosquito-borne alphaviruses belonging to the VEEV, EEEV, WEEV, and SFV complexes. Despite substantial sequence divergence, 3D modeling of the 5′SL suggests a conserved overall fold encompassing the first 25–30 nucleotides of the genome (Figure 6). In general, the 5′SL consists of a 7–9 base pair stem, continuous or containing internal bulges, stabilized predominantly by GC interactions. At the top, the stem terminates in a loop of variable size among different viruses. Comparative analysis indicates that viruses with great predicted thermodynamic stability, such as CHIKV, SFV, VEEV, display long stems and/or high GC content (∆G ≈ −14.4, −8.8 and −8.4 kcal/mol, respectively), features that may be consistent with enhanced protection of the Cap0 structure [49]. The EEEV, which is more sensitive to IFIT1, contains a slightly less stable 5′SL (∆G ≈ −7.8 kcal/mol). Notably, in SINV, the 5′SL is predicted to participate in a pseudoknot interaction that contributes to RNA stabilization. Because this tertiary interaction was not incorporated into thermodynamic predictions, the estimated ∆G (≈−3.7 kcal/mol) is likely underestimated. Overall, despite differences in primary sequence as well as secondary and tertiary interactions within the 5′SL, the conserved 3D topology supports the notion that evolutionary pressure may contribute to preserving structural integrity, likely to maintain functional shielding of the 5′ cap.

Next, we extended our analysis to additional ecological groups. Notably, ancient aquatic alphaviruses also adopt stem–loop structures at the 5′ terminus that closely resemble the mosquito-borne 5′SL, despite variations in positioning (Figure 6). Compared to mosquito-borne alphaviruses, the 5′SL in SESV and SAV is located further downstream of the cap, at 6 and 13 nucleotides, respectively. In SESV, the 5′SL is entirely contained within the 5′UTR, while in SAV it extends partially into the coding region. As previously observed in SINV, the SAV 5′SL is predicted to be stabilized by pseudoknot interactions, which facilitate cap shielding within the overall RNA architecture. The presence of an analogous 5′SL in aquatic alphaviruses is not unexpected, as ISGs constitute part of an ancient antiviral arsenal of vertebrates, preserved across diverse lineages, including fish and marine mammals [62,63,64]. Moreover, IFIT orthologs capable of restricting cap-dependent viral translation have also been described in aquatic vertebrates, suggesting that the apparent structural convergence of the 5′SL element may reflect evolutionary pressures imposed by the interferon system.

Finally, we analyzed the 5′UTR of mosquito-specific alphaviruses. Although the IFN/IFIT system is absent in mosquitoes, the 5′UTR is still predicted to fold into a stem–loop structure at the 5′ terminus. However, the size and architecture of this 5′SL differ from those observed in the other ecological groups. In particular, EILV is predicted to adopt a 5′SL encompassing approximately 40 nucleotides and displaying relatively high predicted stability (∆G ≈ −10.3 kcal/mol). Because IFIT1-mediated restriction is not operative in mosquitoes, alternative selective pressures are likely to shape the architecture of the 5′SL in mosquito-specific alphaviruses. In this regard, it has been proposed that, beyond counteracting the innate immune responses, the 5′SL may function as the promoter for viral RNA synthesis [48,65]. Another possibility is that the 5′SL represents an ancestral structural feature that was retained from progenitor alphaviruses replicating in hosts with functional IFN defenses, despite the apparent relaxation of this selective pressure in mosquito-specific lineages.

Altogether, our analysis suggests that the 5′UTR of most alphaviruses tends to adopt stem–loop structures at the 5′ terminus. These features may reflect a combination of structural constraints and functional requirements, including roles in viral RNA synthesis and potential modulation of host recognition. However, further experimental studies will be required to determine whether these mechanisms are broadly conserved across the genus.

## 8. Single Mutation–Induced Destabilization of the 5′SL Informs the Design of Attenuated Vaccine Candidates

It has been proposed that the G3A substitution in the 5′UTR of VEEV is sufficient to induce a structural rearrangement of the 5′SL, contributing to the increased sensitivity to type I IFN of the attenuated TC-83 strain compared with the wild-type TRD strain [52]. To further explore the impact of this single mutation on RNA folding, we used AlphaFold-based structural modeling to predict the 3D conformation of the 5′SL in the TRD and TC-83 strains. The analysis suggests that the first nucleotides of both viral genomes fold into stem-loop structures; however, their predicted 3D architectures differ in the pattern of nucleotide interactions (Figure 6). In the TRD 5′SL, five GC base pairs are predicted to stabilize the base of the stem, consistent with a more rigid structure. In contrast, these interactions appear reorganized in TC-83, leading to alternative base pairing contacts. Although a stem–loop topology is still preserved, the base of the stem seems to be less stable, with no more than two contiguous nucleotide interactions.

**Figure 7:**
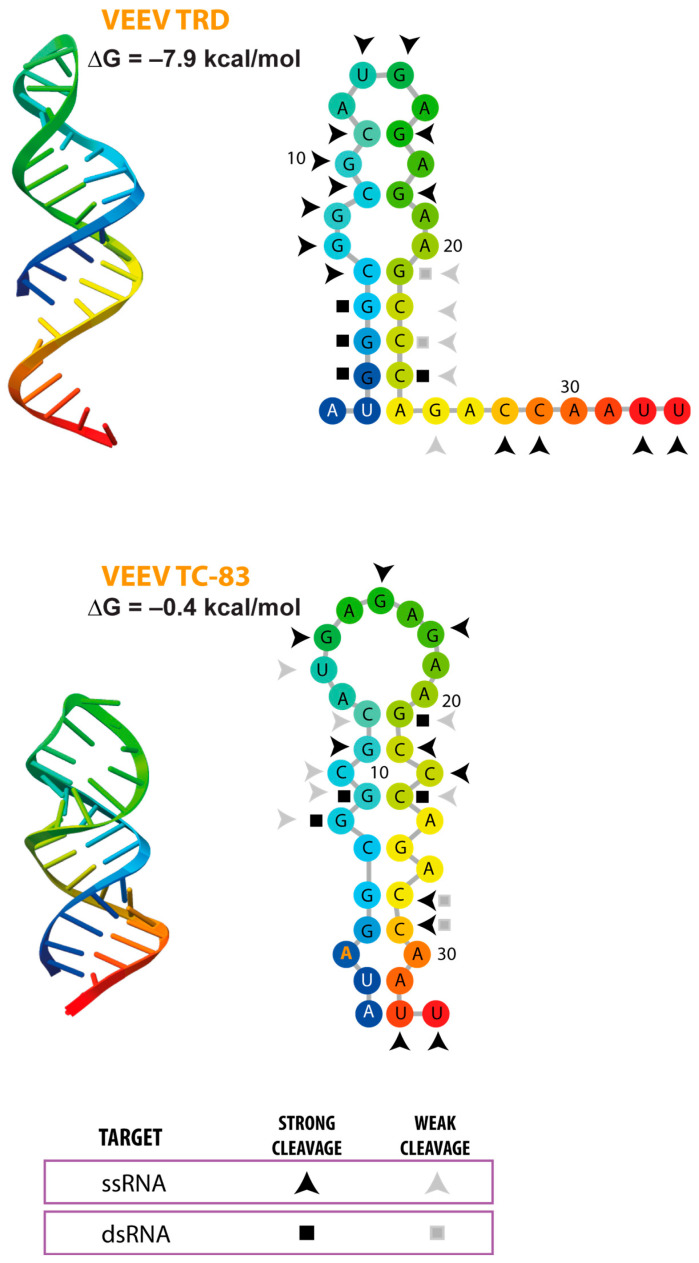
Structural comparison of the 5′SL of two VEEV strains: the wild-type TRD and the vaccine candidate TC-83. Both SLs differ by a single nucleotide: TC-83 carries an A instead of a G at the third position (highlighted in orange). The first nucleotides of each viral genome were folded using the Alphafold algorithm to predict 3D structures. Based on these predictions, RNA secondary structures were modeled with forna. Both 2D and 3D representations are shown using a rainbow color scale, which indicates nucleotide position along the RNA, with the 5′ end depicted in blue and the 3′ end, in red. Thermodynamic stability of each predicted structure was estimated with the RNAeval program. Experimental validation by enzymatic probing, performed by Kulasegaran-Shylini et al. [65], is also shown. ssRNA regions were detected by RNase A (C- and U-specific) and RNase T1 (G-specific) treatments, whereas dsRNA regions were identified by RNase V1 treatment. Strong and weak cleavage sites are indicated.

Enzymatic probing experiments support the AlphaFold predictions, as nucleotide sensitivity to single-strand- and double-strand-specific RNases differs between the 5′SLs of TRD and TC-83, consistent with the proposed 2D structural model (Figure 7). These differences are further reflected in the estimated ΔG values (−7.9 kcal/mol for TRD and −0.4 kcal/mol for TC-83), indicating substantially reduced thermodynamic stability in the TC-83 variant.

The lower stability of the TC-83 5′SL is also consistent with experimental RNA unfolding data obtained by circular dichroism spectroscopy [52]. Temperature-dependent changes in ellipticity revealed multiple transitions, likely corresponding to cooperative unfolding events. Importantly, a prominent transition near 75 °C was observed in the G3 RNA but not in the A3 RNA, supporting differences in thermal stability between these variants.

In summary, current evidence supports the idea that 5′SL structural stability can be modulated to influence susceptibility to IFIT1. Single nucleotide substitutions may be sufficient to destabilize the 5′SL and attenuate viral replication without disrupting other essential functions of this element during the viral life cycle. Together, these observations point to an RNA structure as a determinant of immune evasion and could represent a potential target for rational attenuation strategies.

## 9. Discussion

This review summarizes current evidence on how host factors sense the Cap0 structure in alphavirus genomes and how these viruses evade innate immune detection through the formation of a structured 5′ RNA element, the 5′SL. Although primary sequences differ, diverse alphavirus 5′UTRs adopt conserved stem–loop architectures, supporting the idea that RNA conformation rather than sequence dictates host recognition.

A recent analysis has demonstrated that the CHIKV frameshifting element function is governed by kinetic folding pathways rather than equilibrium stability [66]. In that study, thermodynamic parameters alone failed to predict frameshifting efficiency, as mutants with similar free energy exhibited markedly different functional outcomes. Instead, activity correlated with the formation of long-lived folding intermediates and kinetic traps that limited access to alternative conformations. In light of these observations, the biologically relevant 5′SL RNA structure may not necessarily correspond to the minimum-free-energy conformation, but rather to a structure that is kinetically accessible during folding. Nevertheless, kinetic analyses of the 5′SL are still lacking, and further studies will be needed to determine how RNA folding dynamics—beyond cap chemistry and steady-state structure—may influence PAMPs exposure and the efficiency of immune recognition.

Understanding how RNA features modulate innate immune recognition may have implications for vaccine design, as minimizing unwanted immune sensing while preserving efficient antigen expression remains a central challenge. In the context of live-attenuated vaccines, a delicate balance between viral replication and immune sensing must be achieved: excessive attenuation may reduce immunogenicity, whereas insufficient attenuation may compromise safety [67,68]. Nowadays, mRNA vaccines represent a revolutionary platform for inducing protective immune responses without requiring viral replication [69,70]. These vaccines encode heterologous antigens that are rapidly translated upon entry into host cells, eliciting robust immunity [71]. However, the synthetic nature of in vitro transcribed mRNA can activate innate immune receptors (including RIG-I, MDA5, and IFIT1), potentially reducing antigen expression and promoting excessive inflammation [72]. To mitigate this, mRNA design incorporates specific modifications aimed at limiting detection by PRRs. These include co-transcriptional addition of a Cap1 structure, commonly achieved using technologies such as CleanCap [73], and the incorporation of chemically modified nucleosides, such as pseudouridine and N1-methyl-pseudouridine [74,75]. These analogs retain high-affinity binding to RIG-I but fail to trigger the conformational changes required for downstream signaling [76] (Figure 4). In addition, recent studies have examined the effect of cap-adjacent N6,2′-O-dimethyladenosine (m^6^A_m_) modifications [77] generated by the methyltransferase PCIF1, which catalyzes N6-methylation of the first transcribed adenosine already bearing 2′-O-methylation [78,79]. Importantly, m^6^A_m_ prevents the formation of complexes between IFIT proteins and RNA more effectively than other cap structures, including Cap1 [77]. Collectively, these strategies enable synthetic mRNAs to enhance translational efficiency, ensuring vaccine performance. Such innovations were central to the success of mRNA vaccines against SARS-CoV-2 [80,81] and offer a valuable framework for the development of next-generation vaccines targeting other RNA viruses [82].

Another promising platform under development is based on self-amplifying RNA (saRNA) molecules [83,84]. These constructs are typically derived from alphavirus viral genomes in which the structural protein genes are replaced by a sequence encoding the antigen of interest, while retaining the nonstructural proteins required for RNA replication. The main advantage of using saRNA as a vaccine platform is that high levels of antigen expression can be achieved with substantially lower RNA doses, owing to the replicative capacity of the molecule. Like mRNA vaccines, saRNA platforms must carefully modulate detection by PRRs to ensure efficient antigen expression. In this context, although still speculative, engineering the alphavirus 5′SL may also contribute to shaping innate immune recognition.

As mentioned, beyond its role in immune evasion, the 5′SL has also been implicated in recognition by the viral RNA-dependent RNA polymerase during the synthesis of both negative- and positive-strand RNAs [48,65]. It is therefore possible that the 5′SL has evolved to fulfill dual requirements, contributing to reduced IFIT-mediated recognition while also acting as a promoter element for RNA replication. In this context, some degree of structural variations may be tolerated, provided that these core functions are preserved.

A key limitation in the understanding of the 5′SL structure lies in the lack of high-resolution experimental validation. Moreover, while computational models provide valuable insights into potential folding and stability, they do not capture the full complexity of RNA dynamics in the cellular context. In addition, it remains unclear how the RNA structure is affected by interactions with ligands or associated proteins. Future high-resolution structural studies of the 5′SL, including approaches such as cryo-electron microscopy or X-ray crystallography, particularly in complex with interacting proteins, may help clarify cap positioning relative to IFIT1 and identify RNA features involved in polymerase recognition, thereby informing the rational design of improved vaccine platforms.

RNA viruses have evolved diverse strategies to evade host antiviral responses targeting the 5′ cap structure [85]. Many cytoplasmic RNA viruses, including flaviviruses, coronaviruses, poxviruses, paramyxoviruses, reoviruses, and rhabdoviruses, encode their own 2′-O-methyltransferases to generate Cap1 structures that closely mimic host mRNAs. This strategy provides high translatability and robust immune evasion but entails a genetic investment, as it requires specialized viral enzymes [86,87,88]. Other viruses, such as orthomyxoviruses, bunyaviruses, and arenaviruses, employ a “cap-snatching” mechanism, acquiring Cap1 structures by cleaving them from host mRNAs to ensure perfect molecular mimicry [89]. This approach ensures optimal immune evasion without the need to encode capping enzymes but creates dependence on host transcriptional activity. Alternatively, some positive-strand RNA viruses, including picornaviruses and hepaciviruses, bypass the need for the cap entirely by using internal ribosome entry site (IRES) elements, thus evading cap-dependent restriction factors [90,91]. While cap-independent translation strategies, as well as RNA structure-based mechanisms, may represent more economical solutions, they often involve trade-offs in translational efficiency and dependence on the cellular context [92]. Overall, these strategies illustrate a clear case of convergent evolution in which different viruses have developed alternative yet functionally analogous solutions to minimize detection by host RNA sensors while ensuring efficient translation.

Importantly, the evolutionary success of viral immune evasion strategies is not dependent solely on their intrinsic efficiency against cap-dependent restriction factors but on their integration with additional layers of immune evasion. In this context, several viruses directly antagonize PRR signaling pathways. For example, the NS1 protein of influenza A virus inhibits RIG-I activation [93] and also hijacks YAP/TAZ to suppress TLR3-mediated innate immune responses [94]. In turn, the nsP2 protein of CHIKV inhibits key components of the type I IFN response [95]. In addition, alphaviruses [96] and other RNA viruses [97] replicate within membrane-bound compartments, thereby limiting exposure of their RNA to cytosolic sensors. Taken together, these observations suggest that viral immune evasion is best understood as a multilayered process, in which different mechanisms act in combination to modulate host recognition. This diversity reflects the ongoing evolutionary interplay between host antiviral defenses and viral strategies that mitigate immune detection.

Ultimately, a more integrated understanding of how these mechanisms operate not only illuminates fundamental aspects of virus–host interactions but also offers a powerful framework for the rational design of next-generation vaccine strategies.

## 10. Open Questions

1. While the role of IFIT1 in restricting alphaviruses is well documented, several important questions remain. How does the 5′SL influence recognition by other innate immune receptors, such as RIG-I or TLRs? Structural studies suggest that Cap0 RNAs can bind RIG-I in specific contexts, particularly when dsRNA elements are present [26,27]. However, further investigation is needed to determine whether structured elements in alphavirus RNAs can broadly suppress or delay detection by sensors beyond IFIT1.
2. Viral RNA genomes are highly plastic, adopting alternative conformations at different stages of the replication cycle. It will therefore be important to elucidate how dynamic the 5′SL structure is in vivo, whether it undergoes conformational rearrangements during infection, and how such structural transitions influence its recognition by innate immune sensors.
3. Although the 5′SL 3D structure can be approximated using AI-driven algorithms in combination with SHAPE reactivity data, its precise high-resolution architecture across different alphaviruses remains to be experimentally defined. Moreover, as these predictions were performed on isolated RNA sequences, how interactions with viral and host factors may remodel this structure remains an open question. In addition, a comprehensive characterization of the host interactome associated with the 5′SL is still lacking. Identifying the full spectrum of host factors that bind this element and defining how these interactions influence RNA stability, replication, and immune sensing will be essential to fully understand its multifunctional role during infection.
4. While the 5′SL appears to be conserved across alphaviruses, many functional studies have been performed in a limited number of viral models. It therefore remains unclear whether IFIT evasion mediated by the 5′SL is restricted to specific viruses or represents a more general mechanism shared among alphaviruses that replicate in vertebrate hosts.
5. The impact of a single mutation within the 5′SL on the replicative capacity of VEEV provides an important proof of concept for the potential manipulation of this element in rational vaccine design. To what extent can the stability of the 5′SL be fine-tuned to achieve predictable levels of attenuation without compromising replication competence? Could rational manipulation of RNA structural PAMPs be generalized as a broader strategy for engineering safer and more immunogenic RNA-based vaccine platforms?

## Data Availability

All data are available in the manuscript.

## Supplementary Materials

The following supporting information can be downloaded at: https://www.mdpi.com/article/10.3390/v18040439/s1. Figure S1: Quality of Alphafold-predicted RNA structures.
